# Subretinal timrepigene emparvovec in adult men with choroideremia: a randomized phase 3 trial

**DOI:** 10.1038/s41591-023-02520-3

**Published:** 2023-10-09

**Authors:** Robert E. MacLaren, M. Dominik Fischer, James A. Gow, Byron L. Lam, Eeva-Marja K. Sankila, Aniz Girach, Sushil Panda, Dan Yoon, Guolin Zhao, Mark E. Pennesi

**Affiliations:** 1 Nuffield Laboratory of Ophthalmology, Department of Clinical Neurosciences, University of Oxford, Oxford, UK; 2 Oxford University Hospitals NIHR Biomedical Research Centre, Oxford, UK; 3 Oxford Eye Hospital, Oxford University Hospitals NHS Foundation Trust, Oxford, UK; 4 University Eye Hospital Tübingen, Center for Ophthalmology, Tübingen, Germany; 5 Biogen, Cambridge, MA USA; 6 Bascom Palmer Eye Institute, University of Miami Miller School of Medicine, Miami, FL USA; 7 Helsinki University Eye Hospital, Helsinki, Finland; 8 Formerly of Nightstar Therapeutics, London, UK; 9 Department of Ophthalmology, Casey Eye Institute, Oregon Health & Science University, Portland, OR USA

**Keywords:** Drug development, Pharmacogenetics

## Abstract

Choroideremia is a rare, X-linked retinal degeneration resulting in progressive vision loss. A randomized, masked, phase 3 clinical trial evaluated the safety and efficacy over 12 months of follow-up in adult males with choroideremia randomized to receive a high-dose (1.0 × 10^11^ vector genomes (vg); *n* = 69) or low-dose (1.0 × 10^10^ vg; *n* = 34) subretinal injection of the AAV2-vector-based gene therapy timrepigene emparvovec versus non-treated control (*n* = 66). Most treatment-emergent adverse events were mild or moderate. The trial did not meet its primary endpoint of best-corrected visual acuity (BCVA) improvement. In the primary endpoint analysis, three of 65 participants (5%) in the high-dose group, one of 34 (3%) participants in the low-dose group and zero of 62 (0%) participants in the control group had ≥15-letter Early Treatment Diabetic Retinopathy Study (ETDRS) improvement from baseline BCVA at 12 months (high dose, *P* = 0.245 versus control; low dose, *P* = 0.354 versus control). As the primary endpoint was not met, key secondary endpoints were not tested for significance. In a key secondary endpoint, nine of 65 (14%), six of 35 (18%) and one of 62 (2%) participants in the high-dose, low-dose and control groups, respectively, experienced ≥10-letter ETDRS improvement from baseline BCVA at 12 months. Potential opportunities to enhance future gene therapy studies for choroideremia include optimization of entry criteria (more preserved retinal area), surgical techniques and clinical endpoints. EudraCT registration: 2015-003958-41.

## Abstract (web summary)

In the phase 3 STAR trial, subretinal administration of the AAV2-based gene therapy timrepigene emparvovec in patients with choroideremia did not lead to a significant difference in the primary endpoint of 15-letter Early Treatment Diabetic Retinopathy Study (ETDRS) improvement in visual acuity from baseline compared to untreated controls.

## Main

**Fig. 1:**
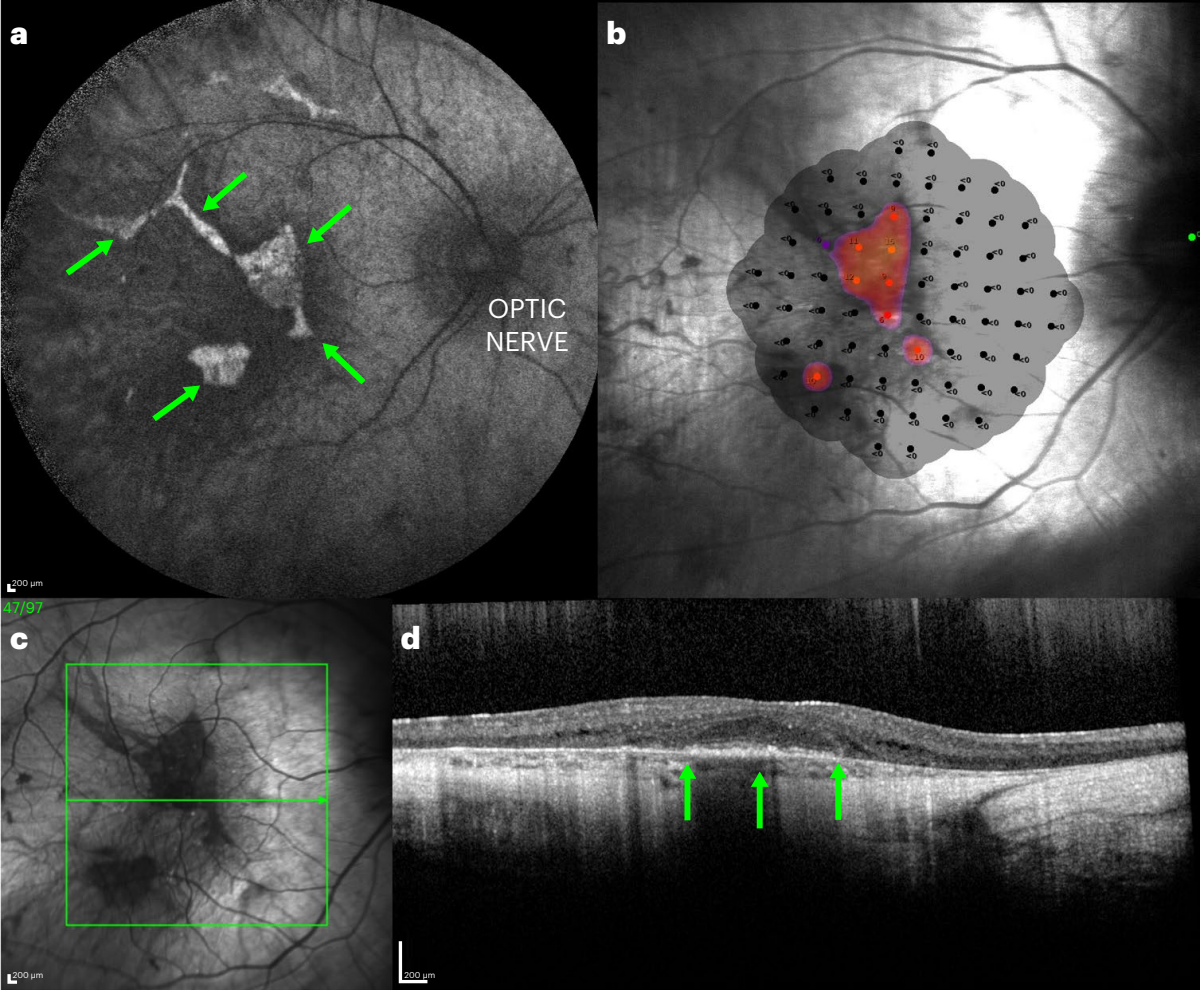
Retinal images and microperimetry plot from a patient with advanced choroideremia. **a**, AF imaging reveals the fluorescent shapes that represent the surviving retinal pigment epithelium centrally (green arrows). **b**, The microperimetry plot shows that the central triangular area of AF is broadly correlated with the surviving visual field (red dots). **c**, The green box shows the region of optical coherence tomography scan, with the scan along the green arrow shown in **d**. **d**, The surviving outer nuclear layer is the area above the green arrows, with disruption of the outer segments indicating early degeneration.

Choroideremia is a rare, X-linked recessive inherited retinal degeneration resulting in progressive vision loss, ultimately leading to blindness^1–3^. The estimated prevalence of choroideremia ranges from 0.5 to 2 per 100,000 people^2,4^. Affected individuals typically demonstrate an early-onset, severe chorioretinal degeneration owing to the X-linked recessive mode of inheritance^5^. Molecular diagnosis is typically required to confirm clinical findings^6^. Recently, it has been observed that splice-site mutations may lead to a milder phenotype when there are very low levels of correctly spliced mRNA; as little as 2–5% of the wild-type transcript levels may significantly attenuate disease progression^7^. The condition is likely underdiagnosed because of its similarities in early stages to other inherited retinal diseases, such as retinitis pigmentosa^2,8^. Choroideremia initially presents in childhood and early adolescence as night blindness^9–11^. Slow and progressive vision loss associated with choroideremia starts from the periphery of the visual field, and best-corrected visual acuity (BCVA) decreases as the disease advances with age^12–14^. In a retrospective study of 71 males with choroideremia, the average age of onset of night blindness symptoms was reported to be 12.6 years (±1.0 year), with loss of peripheral vision at 19.7 years (±1.3 years) (ref. ^9^). Individuals with choroideremia generally retain good central vision until approximately 40 years of age, followed by a rapid reduction of visual acuity in the advanced stage as degeneration starts impacting the fovea (for representative retinal images from a patient with advanced choroideremia, see Fig. 1)^15^. This impairment phase may last 5–10 years until vision is no longer recordable and provides the only potential period during which visual acuity changes might be assessed against a potential treatment^16^.

Choroideremia is caused by mutations in the *CHM* gene, which encodes Rab escort protein 1 (REP1) (refs. ^3,17^). *CHM* mutations decrease REP1 expression, leading to degeneration of the retinal pigment epithelium (RPE), photoreceptors and choroid^3,17^. REP1 serves as a mediator of intracellular trafficking of prenylated Rab proteins in the retina and RPE^3^. Most *CHM* gene mutations responsible for clinical phenotypes cause loss of function either via deletion or nonsense mutations^3^. Missense mutations in the *CHM* gene have also been reported on rare occasions and may result in decreased levels of REP1 expression and protein structure destabilization^3^.

Ocular conditions are optimal for gene therapies. Retinal cells are post-mitotic, enabling sustained gene expression without the need for genomic integration of transgenic material; the blood-ocular barrier facilitates immune privilege, limiting immunological response to gene therapy products; and gene transduction may be achieved at a low dose, potentially reducing manufacturing burden^18,19^. Viral vector–based gene therapy is being widely studied in both preclinical and clinical settings for the treatment of choroideremia and other inherited retinal dystrophies^6,20^. Adeno-associated viral vectors, such as adeno-associated virus serotype 2 (AAV2), have been shown to achieve efficient transduction of photoreceptors and RPE after subretinal injection and have an acceptable safety profile^19,21–24^. The first ocular gene therapy approved by the US Food and Drug Administration (FDA) uses an AAV2 vector delivered via subretinal injection^19^. Clinical data suggest that AAV2 vectors have no long-term retinal toxicity at the subretinal dose range of 1.0 × 10^10^ to 1.0 × 10^11^ vector genomes (vg) and, in addition to high specificity for RPE transduction, may be able to target rod photoreceptors more effectively than some other AAV serotypes. Inclusion of the inactivated woodchuck hepatitis virus post-transcriptional regulatory element (WPRE) may further boost retinal gene expression of the REP1 protein by up to a log unit in some cases^25^. These observations of both safety and tropism efficacy provide the optimal rationale for the AAV2 choroideremia gene therapeutic strategy^19,23^.

Timrepigene emparvovec (BIIB111/AAV2-REP1) is an AAV2 vector–based gene therapy encoding the wild-type *CHM* cDNA sequence driven by the strong ubiquitous CAG promoter and augmented by an inactivated WPRE sequence^26^. By restoring absent REP1 expression, timrepigene emparvovec aims to address the underlying genetic cause of choroideremia^23^. Data from phase 1/2 studies have demonstrated that timrepigene emparvovec–based gene therapy improved visual acuity in a subset of patients with choroideremia who received treatment^26–29^. In most patients after treatment, BCVA in the study eye improved or remained stable, and, for patients with moderate vision loss at baseline (that is, 34–73 Early Treatment Diabetic Retinopathy Study (ETDRS) letters), higher mean gains in vision at 24 months were observed (5.6 letters) compared to all patients (3.1 letters)^30^. Three patients (9%) achieved and maintained a clinically significant gain of ≥15 ETDRS letters at 24 months^30^. Here we report the results from the randomized, parallel-controlled, phase 3 STAR clinical trial that evaluated the efficacy and safety of timrepigene emparvovec versus a non-surgical control in adult males with genetically confirmed diagnosis of choroideremia.

## Results

### Participant disposition and baseline characteristics

**Fig. 2:**
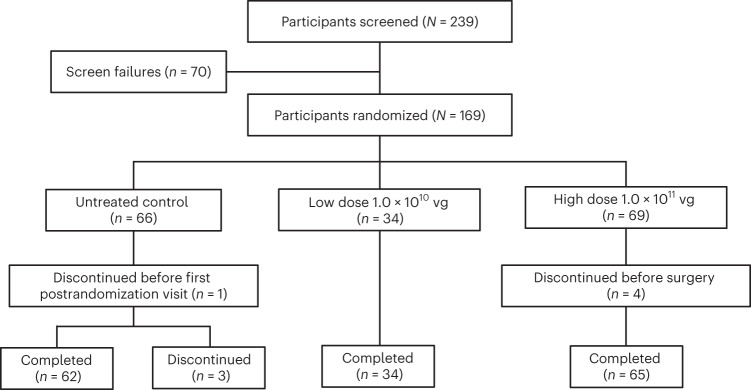
Participant disposition. Flow chart showing pattern of participant recruitment, randomization and follow-up.

A total of 169 participants were randomized (high dose, *n* = 69; low dose, *n* = 34; control, *n* = 66), and 164 participants completed their surgery or attended a post-baseline visit in the study (high dose, *n* = 65; low dose, *n* = 34; control, *n* = 65) (Fig. 2). Demographics were generally balanced across the three study groups (Table 1). Most participants were White, and most were 40–60 years of age. Participants across study groups were well distributed in the study sites located in Finland, Germany, the United Kingdom and the United States.

**Table 1:** Participant demographics and baseline characteristics: safety population

		Timrepigene emparvovec		
	Control group (*n* = 65)	Low dose (*n* = 34)	High dose (*n* = 65)	All participants (*n* = 164)
Age, years^a^				
Mean (s.d.)	49.4 (13.7)	49.8 (12.6)	47.5 (12.9)	48.7 (13.2)
Median (Q1, Q3)	50.0 (40.0, 60.0)	52 (42.0, 60.0)	49.0 (37.0, 57.0)	51.0 (38.5, 59.0)
Sex				
Male, *n* (%)	65 (100)	34 (100)	65 (100)	164 (100)
Ethnicity^b^, *n* (%)				
Hispanic or Latino	3 (4.6)	1 (2.9)	4 (6.2)	8 (4.9)
Not Hispanic or Latino	51 (78.5)	26 (76.5)	54 (83.1)	131 (79.9)
Not reported	11 (16.9)	7 (20.6)	7 (10.8)	25 (15.2)
Race^b^, *n* (%)				
Asian	0 (0)	0 (0)	1 (1.5)	1 (0.6)
American Indian or Alaska Native	1 (1.5)	0 (0)	0 (0)	1 (0.6)
Black or African American	1 (1.5)	0 (0)	0 (0)	1 (0.6)
Native Hawaiian or other Pacific Islander	0 (0)	0 (0)	0 (0)	0 (0)
White	55 (84.6)	30 (88.2)	59 (90.8)	144 (87.8)
Other	1 (1.5)	0 (0)	0 (0)	1 (0.6)
Not reported	7 (10.8)	4 (11.8)	5 (7.7)	16 (9.8)
Baseline weight				
*n*	63	34	65	162
Mean (s.d.), kg	85.3 (16.6)	94.4 (15.4)	90.3 (17.8)	89.2 (17.1)
Median (Q1, Q3), kg	85.0 (73.0, 97.5)	91.0 (84.0, 108.1)	86.6 (76.3, 101.1)	86.4 (77.5, 100.0)
Surgical site, n (%)				
Oxford, UK	13 (20.0)	6 (17.6)	12 (18.5)	31 (18.9)
Tübingen, Germany	14 (21.5)	7 (20.6)	14 (21.5)	35 (21.3)
Miami, FL, USA	15 (23.1)	8 (23.5)	15 (23.1)	38 (23.2)
Portland, OR, USA	16 (24.6)	9 (26.5)	17 (26.2)	42 (25.6)
Helsinki, Finland	7 (10.8)	4 (11.8)	7 (10.8)	18 (11.0)
BCVA, letters, mean (s.d.)				
Study eye	60.4 (8.7)	61.8 (8.1)	58.7 (8.9)	60.0 (8.7)
Fellow eye	59.8 (23.3)	65.3 (21.0)	62.5 (20.4)	62.1 (21.7)
Microperimetry – mean sensitivity, dB, mean (s.d.)				
Study eye	1.59 (2.45)	1.37 (2.78)	1.69 (2.66)	1.58 (2.60)
Fellow eye	2.13 (3.50)	1.74 (2.51)	2.03 (3.20)	2.00 (3.16)
Fundus AF – total area of preserved AF, mm^2^, mean (s.d.)				
Study eye	3.045 (2.764)	3.449 (3.340)	3.165 (3.120)	3.183 (3.028)
Fellow eye	3.724 (4.256)	4.086 (4.652)	3.986 (4.490)	3.908 (4.411)
Pelli-Robson contrast sensitivity score, mean (s.d.)				
Study eye	0.932 (0.366)	0.971 (0.398)	0.945 (0.383)	0.946 (0.378)
Fellow eye	0.998 (0.509)	1.159 (0.435)	1.035 (0.442)	1.048 (0.468)
Color vision test, *n* (%) with defect				
Study eye	50 (96)	26 (90)	53 (100)	129 (96)
Fellow eye	43 (88)	25 (86)	51 (98)	119 (92)
Reading speed, words per minute, mean (s.d.)				
Study eye	322 (1,010)	70 (39)	74 (52)	166 (627)
Fellow eye	486 (1,695)	89 (39)	90 (57)	233 (1,029)

Q1, first quartile; Q3, third quartile.

^a^Age was calculated as the number of years between the date of birth and the informed consent date.

^b^Self-reported.

### Safety

The overall incidence of treatment-emergent adverse events (TEAEs) was generally higher in the timrepigene emparvovec groups (59/65 participants in the high-dose group (91%); 32/34 participants in the low-dose group (94%)) than in the control group (33/65 participants (51%)) (Table 2). No participants died or discontinued from the study because of TEAEs. Most TEAEs were mild or moderate in severity. Ocular TEAEs and severe ocular TEAEs occurred more frequently in the treated participants compared to the control group and more commonly in the study eye than in the fellow eye for treated groups. Ocular inflammation–related TEAEs and visual acuity–related TEAEs were common and occurred more frequently in the treated groups. The incidence (*n* (%)) of ocular inflammation–related TEAEs in the high-dose, low-dose and control groups was 33/65 (51%), 16/34 (47%) and 1/65 (2%), respectively. One participant (2%) in the high-dose group experienced a serious ocular inflammation–related TEAE (non-infective retinitis). Visual acuity reduction events were the most reported serious ocular TEAEs. Three of these visual acuity reduction events were related to the study drug, whereas seven of these events were related to the study procedure. Cataracts as TEAEs were observed more frequently in the high-dose group (9/65 (14%)) and low-dose group (4/34 (12%)) than in the control group (3/65 (5%)). Most participants in each treatment group had no shift in lens opacity grade from baseline to month 12, and changes in the lens opacity in the fellow eye for the same follow-up period for all study groups were similar to those in the study eye of the control group. No clinically meaningful changes in vital sign measurements were observed.

**Table 2:** TEAEs in the safety population^a^

		Timrepigene emparvovec	
	Control group (*n* = 65)	Low dose (*n* = 34)	High dose (*n* = 65)
Overall summary of TEAEs			
Any TEAE, *n* (%), events	33 (51), 78	32 (94), 197	59 (91), 321
Non-ocular TEAE	23 (35), 51	20 (59), 53	29 (45), 62
Ocular TEAE	16 (25), 27	29 (85), 144	58 (89), 259
Study eye	11 (17), 13	29 (85), 122	56 (86), 226
Fellow eye	11 (17), 14	16 (47), 22	23 (35), 33
Any treatment-related TEAE, *n* (%), events	0 (0), 0	28 (82), 119	55 (85), 197
Non-ocular treatment-related TEAE	0 (0), 0	5 (15), 7	6 (9), 9
Ocular treatment-related TEAE	0 (0), 0	27 (79), 112	55 (85), 188
Study eye	0 (0), 0	27 (79), 106	55 (85), 188
Fellow eye	0 (0), 0	5 (15), 6	0 (0), 0
Any serious TEAE, *n* (%), events	10 (15), 13	9 (26), 14	11 (17), 16
Any treatment-related serious TEAE, *n* (%), events	0 (0), 0	5 (15), 7	6 (9), 9
Any TEAE leading to death, *n* (%), events	0 (0), 0	0 (0), 0	0 (0), 0
TEAE severity, *n* (%), events			
Mild	16 (25), 45	17 (50), 160	25 (38), 260
Moderate	12 (18), 25	9 (26), 28	27 (42), 54
Severe	5 (8), 8	6 (18), 9	7 (11), 7
TEAE plausible relationship to study drug/procedure, *n* (%), events			
Yes	0 (0), 0	28 (82), 119	55 (85), 197
Related to study drug	0 (0), 0	3 (9), 3	5 (8), 5
Related to study procedure	0 (0), 0	28 (82), 113	54 (83), 183
Related to both study drug and study procedure	0 (0), 0	2 (6), 2	2 (3), 2
Unknown	0 (0), 0	5 (15), 5	9 (14), 11
No	16 (25), 32	4 (12), 77	4 (6), 121
Participant not treated	17 (26), 46	0 (0), 1	0 (0), 3
Missing	0 (0), 0	0 (0), 0	0 (0), 0
TEAEs by SOC and occurring ≥10% in any group			
Ocular TEAE, *n* (%), events	16 (25), 27	29 (85), 144	58 (89), 259
Conjunctival hemorrhage	0 (0), 0	13 (38), 15	26 (40), 26
Anterior chamber cell	0 (0), 0	14 (41), 17	24 (37), 25
Vitritis	0 (0), 0	10 (29), 10	16 (25), 19
Eye pain	0 (0), 0	6 (18), 8	11 (17), 13
Cataract	3 (5), 5	4 (12), 5	9 (14), 14
Foreign body sensation in eyes	0 (0), 0	3 (9), 3	9 (14), 9
Eye irritation	0 (0), 0	5 (15), 6	8 (12), 8
Conjunctival hyperemia	0 (0), 0	4 (12), 5	7 (11), 8
LLVA decreased	0 (0), 0	2 (6), 3	7 (11), 7
Ocular hyperemia	0 (0), 0	4 (12), 4	7 (11), 8
Visual acuity reduced	9 (14), 9	6 (18), 8	7 (11), 10
Cataract subcapsular	0 (0), 0	4 (12), 6	4 (6), 4
Cardiac disorders, *n* (%), events	2 (3), 2	2 (6), 2	2 (3), 3
Gastrointestinal disorders, *n* (%), events	3 (5), 5	2 (6), 3	4 (6), 5
Infections and infestations, *n* (%), events	10 (15), 16	12 (35), 23	20 (31), 31
Injury or procedural complication, *n* (%), events	3 (5), 6	5 (15), 8	10 (15), 14
Metabolism and nutrition disorders, *n* (%), events	3 (5), 3	0 (0), 0	5 (8), 5
Musculoskeletal and connective tissue disorders, *n* (%), events	5 (8), 6	3 (9), 3	1 (2), 1
Nervous system disorders, *n* (%), events	2 (3), 2	4 (12), 5	9 (14), 11
Headache	0 (0), 0	3 (9), 4	7 (11), 8
Psychiatric disorders	4 (6), 4	4 (12), 4	0 (0), 0
Respiratory disorders	1 (2), 1	2 (6), 2	2 (3), 2
Skin and subcutaneous tissue disorders	1 (2), 1	2 (6), 2	1 (2), 1

LLVA, low luminance visual acuity; SOC, system organ class.

^a^TEAEs were defined as AEs starting on or after the day of the surgery (or, for control group participants, visit 2, day 0). If a participant had multiple events of severity and outcome, then this participant was counted only once in the worst hierarchy in each category. However, participants could have been counted more than once in action taken.

### Primary efficacy measure

#### Proportion of participants with ≥15-letter ETDRS increase from baseline in BCVA at month 12

**Fig. 3:**
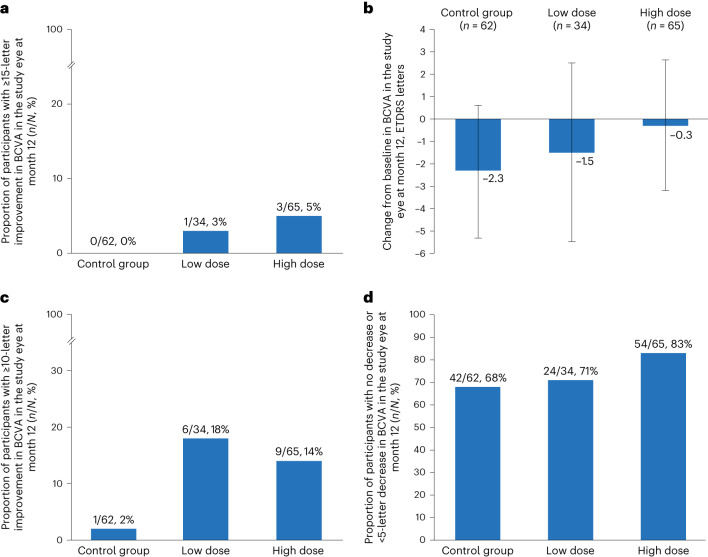
Visual acuity changes in trial participants. **a**–**d**, In all cases, the eyes treated with gene therapy had numerically better outcomes than unoperated control eyes, both in terms of the proportion gaining lines of vision and in the mean changes in visual acuity. Proportion of participants with ≥15-letter ETDRS improvement from baseline (**a**), LS mean change from baseline (**b**), proportion of participants with ≥10-letter ETDRS improvement from baseline (**c**) and proportion of participants with no or <5-letter ETDRS decrease from baseline (**d**) in BCVA (ETDRS letters) in the study eye at month 12 for the intent-to-treat population. LS mean change from baseline was calculated using the ANCOVA model, which includes factors for surgical group and study arms and baseline value of the assessment as covariate. Missing data were imputed by the last observation carried forward approach. Error bars represent 95% CI.

The primary endpoint (proportion of participants with a ≥15-letter ETDRS improvement from baseline in study eye BCVA at 12 months) was not statistically different between high-dose (*n* = 3/65 (5%)) and control (*n* = 0/62 (0%)) groups (*P* = 0.245; Fig. 3a). Although all comparisons for the key secondary endpoints would not be tested (per the hierarchical procedure) if efficacy were not claimed for the primary endpoint, those comparisons were conducted in an exploratory nature. The difference between the proportion of participants in the low-dose group (*n* = 1/34 (3%)) and the control group (*n* = 0/62 (0%)) experiencing ≥15-letter ETDRS improvement from baseline in study eye BCVA at 12 months was also not statistically significant (*P* = 0.354).

The volume of the blebs raised and the degree of reflux of vector into the vitreous would have been variable in these surgically challenging participants, which complicates the simple binary assumption of a low or high dose. Because the low dose is also known to be therapeutic^26^, it is not unexpected to find responders in this group. In a post hoc analysis pooling data from both treatment groups, *n* = 4/99 (4%) treated eyes gained ≥15-letter ETDRS improvement compared to *n* = 0/62 (0%) in the control group.

### Key secondary efficacy measures

#### Change from baseline in BCVA score at month 12

The least squares (LS) mean difference (95% confidence interval (CI)) in the change from baseline in study eye BCVA at month 12 between the high-dose group and the control group was a gain of 2.1 (−2.0, 6.2) ETDRS letters, favoring the high dose (Table 3 and Fig. 3b). The LS mean difference in the change from baseline in study eye BCVA score at 12 months between the low-dose group and the control group was a gain of 0.9 ETDRS letters, favoring the low dose.

**Table 3:** Change from baseline in BCVA at month 12 in the study eye for the intent-to-treat population^a^

		Timrepigene emparvovec	
	Control group (*n* = 62)	Low dose (*n* = 34)	High dose (*n* = 65)
ANCOVA change from baseline BCVA, ETDRS letters			
LS mean (s.e.)	−2.3 (1.50)	−1.5 (2.02)	−0.3 (1.47)
LS mean 95% CI	−5.31, 0.61	−5.47, 2.50	−3.18, 2.64
Difference from control group, mean (s.e.)		0.9 (2.48)	2.1 (2.07)
Difference from control group, 95% CI		−4.04, 5.77	−2.01, 6.17

^a^Missing data were imputed by the last observation carried forward approach. ANCOVA model includes factors for surgical group and study arms and baseline value of the assessment as covariate (degrees of freedom, 153).

#### Proportion of participants with ≥10-letter ETDRS improvement from baseline BCVA at month 12

A greater proportion of participants in the high-dose group (*n* = 9/65 (13.8%)) experienced a ≥10-letter ETDRS improvement from baseline in study eye BCVA at month 12 compared to the control group (*n* = 1/62 (1.6%)) (Fig. 3c). Significant efficacy difference could not be claimed per the prespecified hierarchical testing procedure. A greater proportion of participants in the low-dose group (*n* = 6/34 (17.6%)) experienced a ≥10-letter ETDRS improvement from baseline in study eye BCVA at month 12 compared to the control group (*n* = 1/62 (1.6%)). Considering both treatment groups together, *n* = 15/99 (15%) treated eyes gained ≥10-letter ETDRS improvement compared to *n* = 1/62 (2%) in the control group.

#### Proportion of participants with no decrease or <5-letter ETDRS letter decrease from baseline BCVA at month 12

There was a greater proportion of participants with no decrease from baseline or a decrease of <5 ETDRS letters from baseline in study eye BCVA at month 12 in the high-dose group (*n* = 54/65 (83.1%)) and the low-dose group (*n* = 24/34 (70.6%)) compared to the control group (*n* = 42/62 (67.7%)) (Fig. 3d).

Surgical detachment of the fovea is normally associated with a reduction in visual acuity, as is vector-related inflammation. Hence, the finding that the proportions of participants maintaining at least one line (that is, five letters) of ETDRS acuity in both high-dose (83%) and low-dose (71%) groups were numerically greater than in the unoperated control group (68%) does at least support the safety of the gene therapy treatment.

### Other prespecified secondary efficacy measures

The LS mean differences in the change from baseline to month 12 in the study eye mean retinal sensitivity, bivariate contour ellipse area 63% and bivariate contour ellipse area 95% between the high-dose group and the control group were −0.1573 dB, 0.6606 deg^2^ and −1.4877 deg^2^, respectively. The LS mean differences in the change from baseline to month 12 in the study eye mean retinal sensitivity, bivariate contour ellipse area 63% and bivariate contour ellipse area 95% between the low-dose group and the control group were 0.0478 dB, −0.1610 deg^2^ and −4.4940 deg^2^, respectively. The microperimetry data, however, were generally found to be inconsistent, with most participants unable to perform the test accurately or scoring zero because of the advanced nature of their disease when the degeneration has undermined the fovea^31^.

At month 12, despite a difference in the change from baseline (95% CI) in the study eye total area of preserved autofluorescence (AF) between the high-dose and control groups (−0.1264 mm^2^ (−0.2308, −0.0220)), as well as between the low-dose and control groups (−0.1541 mm^2^ (0.2781, −0.0302)), the treatment groups showed a greater decrease, indicating a worsening condition relative to the control group. The LS mean difference (95% CI) in the change from baseline in the study eye distance from foveal center to nearest border of preserved AF at month 12 was −0.6136 μm (−26.5011, 25.2739) between the high-dose and control groups and 1.8375 μm (−28.8300, 32.5051) between the low-dose and control groups.

At month 12, LS mean differences (95% CI) in changes from baseline in contrast sensitivity score in the study eye did not measurably differ for the high-dose or low-dose group versus the control group (0.0209 deg^2^ (−0.0643, 0.1061) and 0.0465 deg^2^ (−0.0558, 0.1488), respectively). The LS mean difference (95% CI) in the change from baseline in the study eye Color Vision Test Total Error score at month 12 was also unsubstantial for either the high-dose group (19.3775 (−45.8063, 84.5612)) or the low-dose group (8.5912 (−68.9870, 86.1695)) versus the control group. The LS mean difference (95% CI) in the change from baseline in the study eye reading speed at month 12 as compared to the control group was 22.1 (−7.9, 52.1) words per minute for the high-dose group and 27.4 (−7.8, 62.7) words per minute for the low-dose group, and neither difference was noteworthy versus the control group. At month 12, the LS mean difference (95% CI) in the change from baseline in the Visual Function Questionnaire 25 (VFQ-25) composite score was 3.4934 (0.0304, 6.9565) for the high-dose group versus the control group and 4.4207 (0.3095, 8.5319) for the low-dose group versus the control group.

### Alternative data ascertainments due to coronavirus disease 2019

The impact of coronavirus disease 2019 (COVID-19) on usage of alternative data ascertainment and study completion was examined (Extended Data Tables 1 and 2). Overall, 32 participants (19%) had alternative data ascertainment (that is, assessment at local non-study sites or out-of-window visits) at month 12 due to COVID-19 travel restrictions. Most alternative data ascertainments were via extension of out-of-window visits. There were 10 COVID-19-related major protocol deviations, with six involving out-of-window visits and four pertaining to BCVA performed by unmasked assessors. Two participants were seen locally in Brazil because of travel restrictions to the United States. The sensitivity analyses assessing the impact of COVID-19 were conducted for the primary endpoint and the key secondary endpoints. The results were consistent with the primary analyses.

## Discussion

The primary endpoint of a three-line gain was not met in this phase 3 trial. Although a spontaneous gain of three lines of vision was not observed in any of the control group participants, there were not enough participants gaining three lines of vision in the treatment groups to meet statistical significance. However, there were notable observations related to BCVA changes in participants undergoing retinal gene therapy for choroideremia in all visual acuity endpoints tested, including the proportion of participants reporting a two-line gain and mean gain in vision and preservation of at least one line of vision.

A ≥10-letter ETDRS improvement from baseline in BCVA has been considered clinically relevant from the patient’s perspective according to scientific advice given at a workshop by the European Medicines Agency (EMA)^32^. A ≥10-letter ETDRS improvement would be more appropriate for younger patients with better baseline vision and more intact retinal structure who would likely be the most optimal patient for a future gene therapy. Although a three-line gain is typical for FDA approval, it might be challenging to achieve this in many inherited retinal degenerations with significant anatomical damage. This is not the case in age-related macular degeneration or diabetic retinopathy, for instance, as BCVA in these retinal degenerations drops because of fluid leakage in the retina, which can quickly be reversed with drug treatments. Considering that there is no approved treatment for choroideremia, stabilization or any improvement of vision in affected individuals could be considered beneficial.

The proportion of individuals gaining three lines of vision in a meta-analysis of the phase 1/2 choroideremia trials was more than twice the rate observed in this pivotal STAR study^30^. The participants selected for the STAR study, however, had far more advanced disease than those in the earlier trials because of the low BCVA entry requirement. This would limit the BCVA gain potential, and the much thinner retina is likely to be more susceptible to surgically induced damage. This highlights the difficulty in obtaining homogeneous patient groups for clinical trials on rare single-gene retinal degenerations. For future trials, a two-line gain in vision may be a better endpoint given the more advanced stages of disease in participants able to be recruited to a trial because it would be more achievable and yet still likely be noted as a clinically significant change by most clinicians. Furthermore, the potential for a spontaneous two-line gain in the control group may have been less because of their more advanced disease (as was observed).

Although it was not considered for significance per the hierarchical procedure, it is interesting that there was a greater numerical difference in BCVA score from the high-dose group versus control than the low-dose group versus control (Table 3), despite there being a slightly worse mean baseline BCVA for the high-dose group compared to the control group. It is possible that the number of available vector genomes associated with the high dose offset the variability in surgery and dose administration, thus leading to the highest numerical mean gain of ETDRS letters among the three groups.

Although no efficacy claims can be made on the basis of this study, it is interesting to note that the treated eyes underwent iatrogenic retinal detachment in thin and degenerate tissues, which would ordinarily be associated with reduced visual acuity. Instead, there was a trend for improvements in visual acuity to occur more frequently in the high-dose group compared to the control group, albeit not enough to meet statistical significance in this cohort. Because of the low percentage of participants who achieved three lines of visual acuity gain across the study, including none in the control group, a larger study size would be needed to confirm the differences between the treatment groups and control group. Alternatively, the results from this cohort suggest that another trial with a two-line gain as the primary endpoint (if acceptable to the regulators) could be more appropriate for smaller enrollment populations, based on the power calculation derived from the current STAR study data.

The safety profile of timrepigene emparvovec was determined to be acceptable. Most TEAEs in the timrepigene emparvovec treatment groups were related to study procedure rather than to study drug. Most ocular inflammation–related TEAEs occurred within 30 d of study drug administration or surgery and were likely related to the surgical procedure. The occurrence of ocular inflammation–related and visual acuity reduced–related events was not dose dependent. Visual acuity reduction events were the most reported serious ocular TEAEs.

Natural history studies have reported a transition age from slow to rapid BCVA decline of ~39 years^15^. In a cohort of patients with choroideremia who were grouped by age <50 years or ≥50 years, visual acuity was observed not to change significantly in either age group over the 1-year follow-up period^33^. It can, therefore, be difficult to assess improvements over a 12-month period in patients if there is no measurable decline in vision in the control group over this period^34^.

The efficacy of AAV2-based gene therapy in younger patients is unknown. For instance, although improvement in BCVA was not demonstrated, it is possible that preservation of vision with timrepigene emparvovec is more likely to be seen in younger patients who are able to tolerate the risks associated with subretinal gene therapy administration.

To get a large gain in vision, three factors need to be aligned. First, the retina needs to be healthy enough to be able to improve visual acuity by two or more lines; second, the surgery needs to be completed without damaging the retinal architecture; and third, the vector needs to be sequestered at a high enough dose subretinally, without being refluxed back into the vitreous. With the high dose, this is more likely to be achieved, because the third factor is countered by having a log unit higher dose of vector particles. Nevertheless, the other factors remain because there will be some retinas that are simply too advanced to be able to achieve large gains in vision. Similarly, in others, there will be surgical complications that might offset the potential gains. It should be expected, though, that large gains in vision might be seen in some low-dose patients when all three factors go well.

For examining the dose effect more accurately, however, it is more logical to look at the effects on BCVA across the whole cohort because then data from every participant can be included, rather than from only the few who have large BCVA gains. If there were no beneficial effect at all from the vector, then one would predict that the mean BCVA loss from the retinal detachment would be similarly worse in both the high-dose and low-dose groups compared to the control group, because of the negative effects of iatrogenic retinal detachment. The observations of the mean BCVA change from this study, however, are the reverse. Although these values were not formally tested for significance per the hierarchical procedure, the direction and pattern of BCVA changes across the entire participant group with these subanalyses is consistent with a treatment effect that is better with the high dose, especially given that these participants underwent an invasive surgery.

To summarize some previous points in this discussion, this study had several limitations that would be important to address in future trials of gene therapy in choroideremia. COVID-19 resulted in some major protocol deviations in this trial as noted previously, including some out-of-window visits and performance of BCVA measurements by unmasked assessors. However, on the basis of consistent results of analyses of the per-protocol and intent-to-treat populations for the primary endpoint, the protocol deviations did not appear to have a substantial effect on the study outcomes. Additionally, the primary endpoint requiring a three-line gain in BCVA may not have been realistic in this cohort, and a two-line gain should be considered, especially in cohorts with more advanced disease. Participants in the treatment arm also underwent vitrectomy and pre-injection subretinal formation before subretinal injections, inducing a transient and localized detachment of the central retina. Variable success with the surgery could, therefore, potentially be an additional confounder for the treatment effect in participants with advanced choroideremia, as there may have been only a small preserved retinal area for vector delivery, which might not have been accurately targeted in every case^35^. Furthermore, the small preserved retinal area may not be normal given that BCVA is reduced and the area may not tolerate subretinal injection well compared to intervention in milder disease with better-preserved retinas^35,36^. Lastly, given the advanced disease of the participants in the STAR study, the microperimetry data were largely unreliable because patients need reasonably good BCVA and stable fixation to be able to perform the test accurately. There has been a recent uptake in the use of microperimetry in interventional retinal disease trials, including in choroideremia, and it has potential to succeed in a future trial as a clinical endpoint for earlier-stage patients^34^.

Although the primary endpoint was not met in this pivotal trial, gene therapy for choroideremia remains a promising therapeutic approach. Furthermore, visual acuity gains lower than the prespecified threshold for primary endpoint can still be clinically meaningful. However, for advanced disease with a rapid decline in visual acuity, perhaps a more realistic expectation may be to slow down progression and prevent total blindness rather than to improve vision. Potential opportunities to enhance future gene therapy studies for choroideremia include selection of participants with a more preserved retinal area and optimization of surgical techniques and clinical endpoints.

## Methods

### Trial design

**Extended Data Fig. 1:**
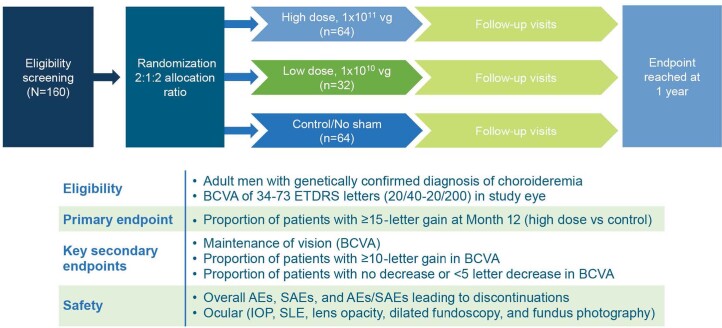
Study design for the STAR phase 3 trial. IOP, intraocular pressure; SLE, slit lamp examination.

The STAR study was prospectively registered in EudraCT as 2015-003958-41 on 16 March 2016 and assigned the identifier NCT03496012 by ClinicalTrials.gov. This investigation was a prospective, randomized, parallel-controlled, outcomes assessor–masked, interventional phase 3 clinical trial that investigated the efficacy and safety of a single subretinal injection of timrepigene emparvovec (BIIB111 or AAV2-REP1) in adult men with choroideremia over the course of eight visits in a 12-month evaluation period (Extended Data Fig. 1). The sequence of timrepigene emparvovec is shown in Extended Data Table 3. The first participant was enrolled on 11 December 2017; the last participant was enrolled on 4 October 2019; and the study was completed on 1 December 2020. Upon discharge of the final participant, the STAR trial concluded. Each participant was assessed for eligibility at their first study visit (visit 1). If they had one eligible eye, that eye was designated the ‘study eye’, and the other, non-eligible eye was designated the ‘fellow eye’. In participants with two eligible eyes, selection of the study eye and fellow eye was made on clinical grounds, and generally the worse eye was assigned to be the study eye; participant choice for study eye was considered in scenarios in which degeneration was relatively symmetrical.

Participant randomization into a treatment or control group occurred at visit 1, during which time a surgical date was scheduled (visit 2) for participants in the treatment group. The requirement of a vitrectomy for administration of the vector meant that a sham treatment was not ethically feasible, and all members of the control group were instead given dates for ‘projected’ surgical visits. This ethical consideration meant that the sponsor, investigator and participants were all unblinded to the surgical procedure for the treatment group; however, for the two doses of timrepigene emparvovec (1.0 × 10^11^ vg (high dose) or 1.0 × 10^10^ vg (low dose)) administered within the treatment group, all parties were blinded as to which dose was received. Randomization ratios were 2:1:2 for the high-dose, low-dose and untreated control groups, respectively. A standard blocked randomization performed by an automated validated system was used for the random assignment of treatment and control groups. After randomization, a change in study eye designation was not permitted. To further minimize potential bias in results, all subjective ophthalmic assessments at visit 1 and visits 5–8 were conducted by a masked assessor.

Visit 2, the projected or actual surgical visit, corresponded to day 0 of the study timeline and took place no later than 8 weeks after visit 1. Visits 3 and 4 were postoperative follow-up visits on day 1 and day 7 that were conducted in-person for participants in a treatment group and by telephone contact for participants in the control group. All participants, regardless of treatment randomization, were planned to attend visits 5–8 on-site, which were scheduled for 1 month, 4 months, 8 months and 12 months after visit 2, respectively. Participants were considered to have completed the study after their eighth study visit.

The investigators (listed in Supplementary Table 1) obtained approval for the study protocol from the appropriate institutional review boards (IRBs) and ethics committees (listed in Supplementary Table 2). The IRBs and ethics committees (principal investigators) were as follows: the University of British Columbia Clinical Research Ethics Board (Kevin Gregory-Evans); McGill University Health Centre REB (Robert Koenekoop); WIRB (Byron Lam, David Birch, Kimberly Stepien and Robert Sisk); Columbia University IRB (Stephen Tsang); Johns Hopkins Medicine IRB (Mandeep Singh); Oregon Health and Science University IRB (Mark Pennesi); UCLA IRB (Michael Gorin); Helsinki and Uusimaa Ethics Committee (Eva-Marja Sankila); the Central Committee on Research Involving Human Subjects (Carel Hoyng); the National Committee on Health Research Ethics (Michael Larsen); CPP South Mediterranean V Ethics Committee (Isabelle Meunier); the Ethics Committee at the Faculty of Medicine of the Eberhard-Karl University and at Tübingen University Hospital (Dominik Fischer and Frank Holz); and London–West London and GTAC Research Ethics Committee (Robert MacLaren and Assad Jalil). Eighteen study sites in North America and Europe received regulatory and local approval for study participation, with 17 sites enrolling participants. Written informed consent was obtained from each participant, and trial conduct was consistent with the United States Code of Federal Regulations, the European Union Clinical Trial Directive and International Council for Harmonisation Good Clinical Practice (E6) and Declaration of Helsinki guidelines.

### Study amendments

There were four protocol amendments. Amendment 1 occurred on 10 November 2015, and it removed treatment of timrepigene emparvovec of the fellow eye that was originally planned for 4–6 participants. Also, it removed the requirement for conducting the International Reading Speed Test in countries where validated translations were not available. Amendment 2 occurred on 26 February 2016 and led to the following changes:

- Changed volume of timrepigene emparvovec subretinal injection from 0.05 ml to 0.1 ml (containing 1 × 10^11^ vg)
- Changed visual acuity inclusion criterion for the study eye from a BCVA of 34–78 letters to a BCVA of 34–73 letters
- Removed randomization method for selection of the ‘study eye’ and replaced with a requirement for the investigator to use clinical judgment (in collaboration with the participant) to select the study eye, which was generally the worse eye
- Clarification of management of screening identification and inclusion of screen failure data
- Removed reference to an Interactive Voice/Web Response System for purposes of treatment randomization
- Included prednisone (in addition to prednisolone) as the corticosteroid of choice in the 21-d perioperative period
- Added requirement that participants must have had a genetically confirmed diagnosis of choroideremia before the screening visit (visit 1)
- Visit windows for visits 7, 8 and 9 decreased from ±21 d to ±14 d

Amendment 3 occurred on 1 August 2017 and included an updated title to reflect changes in the study design; a change in choice in study control and randomization to a parallel, untreated control three-arm study design (high dose, low dose and untreated control); an increase in sample size from 100 to 140 participants; a change to the primary endpoint from improvement of 10 ETDRS BCVA letters to 15-letter improvement; and a change to the key secondary endpoint to use the NIGHT study as a historical control. Amendment 4 occurred on 15 March 2019 and included an increase of sample size to 160 participants; a change of the key secondary endpoint from a historical comparison to the NIGHT study to prospective within-study assessments; the addition of a risk–benefit assessment to clarify vision loss as a known possible adverse event (AE) (and definition of serious adverse events (SAEs) associated with vision loss), therefore precluding it from Suspected Unexpected Serious Adverse Reaction (SUSAR) reporting; and the definition of day 0 for untreated participants to assure that the duration of follow-up was equal for both treated and untreated participants.

### Participant eligibility

#### Inclusion criteria

Participants were male (assigned by the investigators), ≥18 years of age and willing and able to provide informed consent. Participants could be of any race or ethnicity; they self-reported this information; and they were not obligated to disclose it. Because of occasional clinical misdiagnosis of choroideremia, participants must also have had a documented, genetically confirmed diagnosis of choroideremia before randomization. Participants must have had active disease clinically visible within the macular region in the study eye and a BCVA of 34–73 ETDRS letters (worse than or equal to 6/12 or 20/40 Snellen acuity, but better than or equal to 6/60 or 20/200 Snellen acuity in the study eye) to be eligible for participation in the trial. This range excluded both the participants for whom visual acuity readings were so poor that they became unreliable and participants for whom visual acuity readings were so good that there would be no potential for a three-line gain without hitting a ceiling of 6/5 or 20/16 Snellen acuity.

#### Exclusion criteria

Participants were not eligible for study participation if they had a history of amblyopia in the eligible eye or were unwilling to use barrier contraception methods or abstain from sexual intercourse for 3 months if treated with timrepigene emparvovec, as is standard for gene therapy trials. Furthermore, participants should not have had a previous intraocular surgery in the study eye within 3 months of the first visit or any significant ocular or non-ocular disorder that, in the opinion of the investigator, might put the participant at risk, influence the results of the study or affect the ability of the individual to participate in the study. This includes, but was not limited to, individuals with a contraindication to an oral corticosteroid (for example, prednisolone/prednisone), with a clinically significant cataract and who, in the clinical opinion of the investigator, was not an appropriate candidate for subretinal surgery. To be eligible for participation, individuals should also not have taken part in another research study involving an investigational product in the past 12 weeks or received a gene or cell therapy at any time in the past.

Individuals with advanced choroideremia can have variable BCVA readings when the disease causes partial collapse of the fovea—a concept referred to as ‘foveal splitting’ by the investigators. For this reason, participants were recruited from the natural history of choroideremia (NIGHT) study group (ClinicalTrials.gov: NCT03359551), which allowed identification and exclusion of individuals with variable BCVA. Individuals from the NIGHT study whose baseline value at visit 1 was ≥10 letters different in the study eye compared to the previous NIGHT study visit, as well as all individuals who were not recruited from the NIGHT study, underwent three baseline BCVA readings, with the highest reading selected to determine eligibility for the STAR study. At least two of the three values were required to meet eligibility requirements, and the difference between the three assessments could not be ≥10 letters. A BCVA reading was not repeated for those recruited from the NIGHT study whose BCVA on day 1 was <10 letters different from the previous NIGHT study visit. Several individuals who had stable BCVA in one eye but variable readings in the fellow eye could still be recruited into the trial but only with the stable BCVA eye. Others who had a fellow eye outside the trial inclusion criteria range of BCVA were also recruited. Hence, although these individuals were entered into the STAR study for the stable eye, the asymmetric nature of the end-stage choroideremia in a large proportion of participants meant that the fellow eye was not a suitable control. For this reason, the randomization after recruitment included a non-operated control group.

### Interventions and cohorts

Participants were randomized in a 2:1:2 ratio at baseline to receive a volume of up to 100 µl subretinally of a high dose of timrepigene emparvovec (1.0 × 10^11^ vg), a low dose of timrepigene emparvovec (1.0 × 10^10^ vg) or no treatment. The dose range of vector employed was based on previous clinical trials using the AAV2 vector with a chicken β-actin promoter^26,37^ and investigator-driven clinical studies in which AAV2-REP1 was administered to patients with choroideremia^26–28,38^. For individuals randomized to receive treatment, timrepigene emparvovec was administered at the planned dose as a subretinal injection targeting the preserved retinal region of the macula via vitrectomy and after formation of a subretinal bleb using balanced salt solution on the surgical date (day 0) (ref. ^39^). Participants who received timrepigene emparvovec were given a 21-d course of oral corticosteroid to prevent potential inflammation resulting from surgery and immune responses, beginning 2 d before the study dose.

### Endpoints

#### Primary and key secondary endpoints

The primary endpoint of the study was the proportion of participants with a ≥15-letter improvement (amended from ≥10-letter improvement, in accordance with US regulatory requirements) from baseline in BCVA at 12 months as measured by the ETDRS chart. The key secondary endpoints were the mean change from baseline in BCVA at 12 months measured by the ETDRS chart, the proportion of participants with a ≥10-letter ETDRS improvement from baseline in BCVA and the proportion of participants with no decrease in BCVA from baseline or a decrease of <5 ETDRS letters from baseline at 12 months.

#### Safety endpoints

The safety-related assessments included overall AEs, SAEs and AEs or SAEs leading to discontinuations from the clinical trial.

#### Additional secondary endpoints

Other secondary endpoints included the change from baseline to month 12 in the following measures: BCVA, total area of preserved AF, area of preserved ellipsoid zone, microperimetry, contrast sensitivity score, Color Vision Total Error score, reading speed and VFQ-25 score. Change of BCVA from baseline at months 4 and 8 were also secondary endpoints, but the results were not included in this report. Fundus AF was performed to evaluate the changes in the area of viable retinal tissue. Contrast sensitivity was measured before pupil dilation using a Pelli-Robson chart. Color vision was tested separately before pupil dilation. The International Reading Speed Texts (IReST) was used to evaluate reading speed. Self-reported vision-targeted health status responses (individual, subscale and overall composite scores) were obtained using the VFQ-25 questionnaire.

### Statistical methods

#### Sample size

Sample size estimation was performed using Fisher’s exact test. Considering that choroideremia is a degenerative disease, it was assumed that a ≥15-letter BCVA gain would not be observed in participants without treatment. Assuming that 16.7% of the treated participants would gain ≥15 letters in BCVA at 12 months, 56 participants in the high-dose group and the control group would provide ≥90% power at a 0.05 level of significance with a two-sided test. To be conservative, 64 participants in the high-dose group and 64 participants in the control group were needed to ensure 85% power in case one participant in the untreated control group had ≥15-letter BCVA gain by chance, which corresponded to a total of 160 participants completing the study (64 participants in the high-dose group, 32 in the low-dose group and 64 in the control group).

#### Analysis of outcomes

All analyses and summaries were produced using SAS version 9.4 or higher. Primary and key secondary efficacy endpoints were tested under a hierarchical procedure to maintain the type I error for the comparison between the high-dose group and the control group. Nominal *P* values were calculated for comparisons of the high-dose group or low-dose group versus the control group, with a prespecified threshold of significance set at 0.05. Statistical tests and 95% CIs were two-sided. The primary efficacy endpoint was tested first, and, if the *P* value was less than 0.05, then the key secondary endpoints would be tested in the following prespecified order: change from baseline in BCVA at month 12, proportion of participants with a ≥10-letter improvement from baseline in BCVA at month 12 and proportion of participants with either no decrease or a <5-letter decrease from baseline in BCVA at month 12.

Analysis of the primary endpoint was based on the intent-to-treat population, defined as all participants who were randomized, completed visit 2 (that is, received the study treatment or received a phone call (if in control group)) and had at least one post-treatment BCVA measurement. Change from baseline BCVA score was compared between the study groups (that is, high dose versus control and low dose versus control) using Fisher’s exact test supported by a Fisher’s exact Boschloo test with a Berger–Boos correction of beta = 0.001, in which the reported *P* value was two times the one-sided *P* value to maintain the test at 0.05 two-sided level. Results were further described over time using summary statistics for categorical data, including counts, percentages and 95% CI. Missing data were imputed as failures.

Key secondary efficacy endpoints were also based on the intent-to-treat population. Change from baseline in BCVA at 12 months, a key secondary endpoint, was summarized over time using descriptive statistics (that is, mean, s.d. and 95% CI) and evaluated by analysis of covariance (ANCOVA) with missing data handled by the last observation carried forward approach. Fisher’s exact test was used to analyze the proportion of participants with a ≥10-letter improvement from baseline in BCVA at month 12 measured by the ETDRS chart and the proportion of participants with no decrease from baseline in BCVA or a decrease from baseline in BCVA of <5 ETDRS letters at 12 months, respectively.

#### Impact of COVID-19 on statistical analyses

The number and percentage of participants who died during the study due to COVID-19; who withdrew from the study due to COVID-19; whose month 12 visit was performed by an alternative data ascertainment methodology due to COVID-19 (that is, assessment at a local non-study site or extension of the protocol-defined window to include out-of-window visits); and whose month 12 visit was still missed due to COVID-19 despite the opportunity for alternative data ascertainment were summarized by treatment and by the overall cohort. Individuals who had an alternative data ascertainment or missed the month 12 visit entirely were documented as protocol deviations.

For statistical analyses of all BCVA-related endpoints, available alternative data ascertainments were used as month 12 assessments unless an ad hoc visit was made by the participant to the protocol-defined study site within 30 d of the alternative assessment, in which case the data gathered from the ad hoc visit was used. Any instance in which a participant failed to complete the month 12 visit due to withdrawal or death was categorized as missing data and handled the same as other instances of missing data. The sensitivity analyses excluded participants who withdrew from the study or died due to COVID-19, used data from ad hoc visits where available and used imputed data based on alternative data ascertainment visits if the final on-site visit occurred more than 2 months before or 3 months after the planned observation window. These data imputations and/or data handling considerations superseded any subsequent data imputation described above.

### Reporting summary

Further information on research design is available in the Nature Portfolio Reporting Summary linked to this article.

## Online content

Any methods, additional references, Nature Portfolio reporting summaries, source data, extended data, supplementary information, acknowledgements, peer review information; details of author contributions and competing interests; and statements of data and code availability are available at 10.1038/s41591-023-02520-3.

### Supplementary information

Supplementary Information Supplementary Tables 1 and 2.

Reporting Summary

## Figures and Tables

**Extended Data Table 1:**
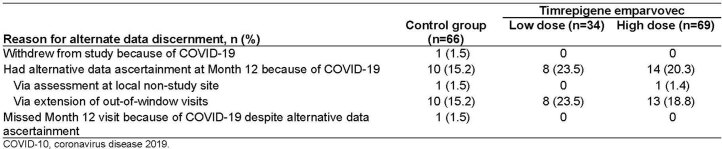
Impact of COVID-19 on patient disposition among all randomized participants

**Extended Data Table 2:**
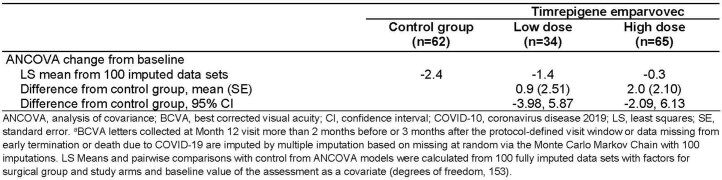
Sensitivity analysis of COVID-19 impact on change from baseline in BCVA in study eye at month 12 for the intent-to-treat population^a^

**Extended Data Table 3:**
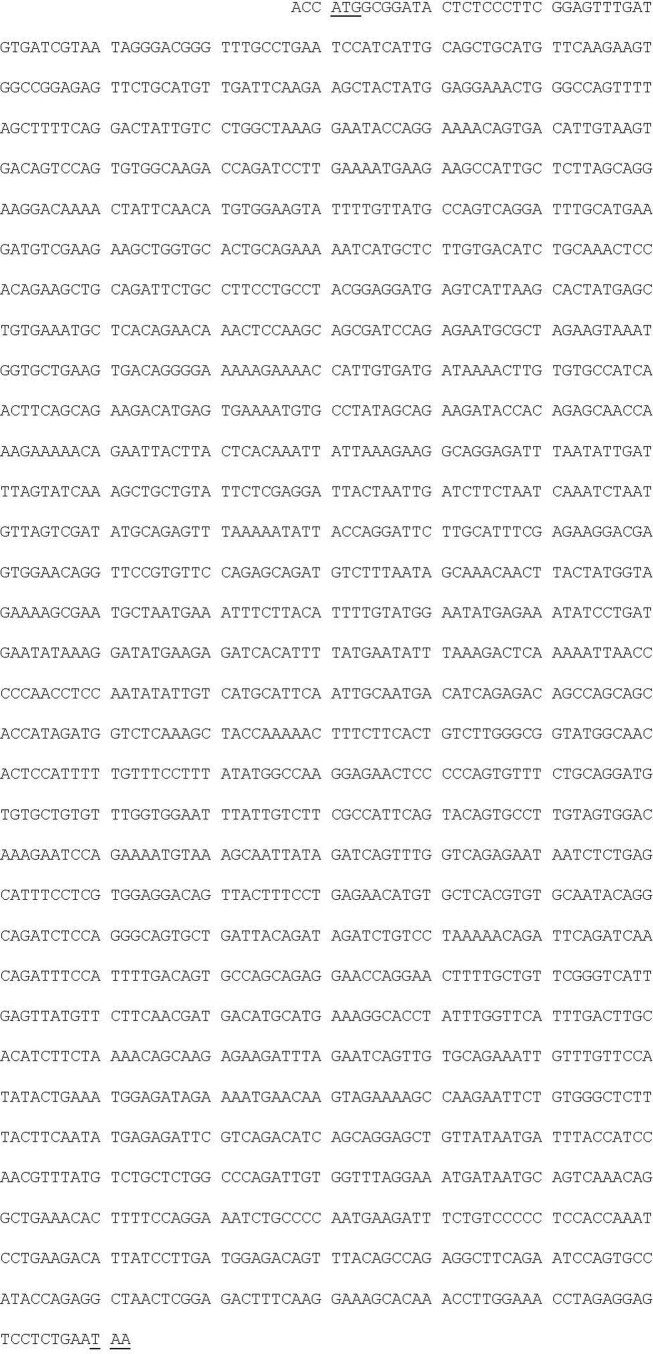
Human *REP1* cDNA sequence used in the rAAV2-REP1 vector (start and stop codons are underlined)

## Data Availability

Trial results are publicly accessible at the EudraCT website (https://www.clinicaltrialsregister.eu/ctr-search/trial/2015-003958-41/results). To request access to additional data, visit https://vivli.org. Individual participant data collected during the trial and that support the research proposal will be available to qualified scientific researchers in accordance with Biogen’s Clinical Trial Transparency and Data Sharing Policy, which is available at https://www.biogentrialtransparency.com. Data requests are initially reviewed by Vivli and Biogen for completeness and other parameters and are then reviewed by an independent review panel. De-identified data and study documents will be shared under agreements that further protect against participant re-identification, and data will be provided in a secure research environment further protecting participant privacy.
