# Supplementary material for: Subretinal timrepigene emparvovec in adult men with choroideremia: a randomized phase 3 trial
Source: Nat Med. 2023 Oct 9;29(10):2464–72. doi: 10.1038/s41591-023-02520-3 (PMC10579095; doi:10.1038/s41591-023-02520-3)
Supplement: Supplementary file 1 — Supplementary Tables 1 and 2. [file 41591_2023_2520_MOESM1_ESM.pdf]

nature medicine

Article

https://doi.org/10.1038/s41591-023-02520-3

Subretinal timrepigene emparvovec in adult men with choroideremia: a randomized phase 3 trial

In the format provided by the authors and unedited

Supplementary Table 1. List of Study Site Investigators and Locations

**Study Site Investigators**	**Site Location**
Kevin Gregory-Evans	The University of British Columbia<br>Eye Care Centre University of British Columbia<br>Retina Research Group<br>VCHA Eye Care Centre<br>Vancouver, Canada
Robert K. Koenekoop	McGill University Health Centre Montreal Children's Hospital<br>Centre for Innovative Medicine (CIM)<br>Montreal, Canada
M. Dominik Fischer<br>Karl Ulrich Bartz-Schmidt<br>Christoph Deuter<br>Rebecca Dollinger<br>Guy Alex Ochakovski<br>Tobias Peters<br>Felix Reichel<br>Paul Richter<br>Yousof Vaheb<br>Barbara Wilhelm<br>Fabian Wozar	University Eye Hospital Tübingen<br>Institute for Ophthalmic Research<br>Centre for Ophthalmology<br>Tübingen, Germany

Supplementary Table 1. List of Study Site Investigators and Locations

Study Site Investigators	Site Location
Frank G. Holz<br>Johannes Birtel<br>Amelie Clemens<br>Philipp Herrmann<br>Philipp L. Müller<br>Katharina Reinking<br>Desiree Völker<br>Peter Charbel Issa	University of Bonn<br>Bonn, Germany
Robert E. MacLaren<br>Thomas M.W. Buckley<br>Thomas L. Edwards<br>Kirti M. Jasani<br>Jasleen Kaur Jolly<br>Jasmina Cehajic-Kapetanovic<br>Moreno Menghini<br>Anika Nanda<br>Salwah Rehman<br>Kanmin Xue<br>Imran H. Yusuf	Oxford Eye Hospital<br>John Radcliffe Hospital<br>Oxford University Hospitals NHS Foundation Trust<br>Oxford, United Kingdom
Assad Jalil<br>Paulo E. Stanga<br>Paul N. Bishop<br>Susmito Biswas<br>Graeme C. M. Black<br>Muhannd El-Faouri<br>Tsveta Ivanova	Manchester Royal Eye Hospital<br>Manchester University NHS Foundation Trust<br>Manchester, United Kingdom

Supplementary Table 1. List of Study Site Investigators and Locations

**Study Site Investigators**	**Site Location**
Byron L. Lam<br>Potyra R. Rosa, Adriana P. Drada, Belen Rodriguez<br>Janet L. Davis<br>Ninel N. Gregori<br>Carlos E. Mendoza-Santiesteban	Bascom Palmer Eye Institute<br>University of Miami Miller School of Medicine<br>Miami, Florida, USA
Stephen H. Tsang<br>Stanley Chang<br>Elona Gavazi<br>Tongalp H. Tezel	Columbia University Irving Medical Center<br>New York, New York, USA
Mandeep S. Singh<br>Mohamed Ahmed<br>Neil M. Bressler<br>Peter A. Campochiaro<br>Mary Frey<br>Shazia Khan<br>Syed Mahmood Shah	Wilmer Eye Institute<br>The Johns Hopkins Hospital<br>Baltimore, Maryland, USA
Mark E. Pennesi<br>Andreas K. Lauer<br>Richard G. Weleber<br>Paul Yang<br>Steven T. Bailey<br>Christina Flaxel	Casey Eye Institute<br>Oregon Health & Science University<br>Portland, Oregon, USA, supported by an NIH (Bethesda, MD) P30 EY010572 core grant, the Malcolm M. Marquis, MD Endowed Fund for Innovation, and an unrestricted grant from Research to Prevent Blindness (New York, NY) to Casey Eye Institute, Oregon Health & Science University.

Supplementary Table 1. List of Study Site Investigators and Locations

**Study Site Investigators**	**Site Location**
David G. Birch<br>Ashkan Abbey<br>Rajiv Anand<br>Deborah Chong<br>Lori Coors<br>Gary E. Fish<br>Rand Spencer<br>Robert C. Wang	Retina Foundation of the Southwest<br>Dallas, Texas, USA
Michael B. Gorin	Jules Stein Eye Institute<br>University of California, Los Angeles<br>Los Angeles, California, USA
Kimberly E. Stepien<br>Kristine Dietzman<br>Melanie A. Schmitt<br>Nickie Stangel	Department of Ophthalmology and Visual Sciences<br>DELETED<br>University of Wisconsin–Madison<br>Madison, Wisconsin, USA
Robert A. Sisk<br>Robert E. Foster<br>Lucas Lindsell<br>Daniel Miller<br>James Osher<br>Christopher Riemann	Cincinnati Eye Institute<br>Cincinnati, Ohio, USA

Supplementary Table 1. List of Study Site Investigators and Locations

Study Site Investigators	Site Location
Eeva-Marja K. Sankila<br>DELETED<br>Henrik Bygglin<br>DELETED<br>Ilkka Immonen<br>Antti Riikonen<br>Sanna Seitsonen	Helsinki University Central Hospital Outpatient Clinic for Hereditary Eye Diseases<br>Helsinki, Finland
Carel Hoyng<br>Niels Crama<br>Jeroen Klevering<br>Dyon Valkenburg	Radboud University Medical Center<br>Nijmegen, Netherlands
Michael Larsen<br>Morten Dornonville de la Cour<br>Joano Duarte<br>Jens Folke Kiilgaard<br>Michael Møller-Hansen<br>Anne-Sofie Petri<br>Simon Paul Rothenbuhler<br>Milkos Schneider<br>Jakob Ørskov Sørensen	Department of Ophthalmology, Rigshospitalet<br>Glostrup, Denmark
Isabelle Meunier<br>Magali Beltran<br>Delphine Jeanjean Nathalie Muenier	CHRU Montpellier Hospital Saint Eloi<br>Montpellier, France

Supplementary Table 2. List of Institutional Review Boards and Ethics Committees

Country	Site	Principal Investigator	Institutional Review Board or Ethics Committee	Central/ Local
Canada	1002	Kevin Gregory-Evans	The University of British Columbia Clinical Research Ethics Board	Local
Canada	1003	Robert Koenekoop	McGill University Health Centre REB	Local
US	4001	Byron L. Lam	WIRB	Local
US	4002	Stephen H. Tsang	Columbia University IRB	Local
US	4003	Mandeep Singh	John Hopkins Medicine IRB	Local
US	4004	Mark E. Pennesi	Oregon Health and Science University IRB	Local
US	4005	David Birch	WIRB	Central
US	4006	Michael B Gorin	UCLA IRB	Local
US	4008	Kimberly Stepien	WIRB	Central
US	4017	Robert Sisk	WIRB	Central
Finland	5001	Eeva-Marja K. Sankila	HELSINKI AND UUSIMAA Ethics Committee	Central
Netherlands	6001	Carel Hoyng	The Central Committee on Research Involving Human Subjects (CCMO)	Central
Denmark	7001	Michael Larsen	The National Committee on Health Research Ethics	Central
France	8001	Isabelle Meunier	CPP SOUTH MEDITERRANEAN V ETHICS COMMITTEE	Central
Germany	2001<br>2002	M. Dominik Fischer<br>Frank Holz	The Ethics Committee at the Faculty of Medicine of the Eberhard-Karl University and at Tübingen University Hospital	Central
UK	3001<br>3003	Robert E. MacLaren<br>Assad Jalil	London - West London and GTAC Research Ethics Committee	Central
