# Supplementary material for: Subretinal timrepigene emparvovec in adult men with choroideremia: a randomized phase 3 trial
Source: Nat Med. 2023 Oct 9;29(10):2464–72. doi: 10.1038/s41591-023-02520-3 (PMC10579095; doi:10.1038/s41591-023-02520-3)
Supplement: Supplementary file 2 — Reporting Summary [file 41591_2023_2520_MOESM2_ESM.pdf]

nature portfolio

Corresponding author(s): Robert E. MacLaren, MD

Last updated by author(s): July 18, 2023

Reporting Summary

Nature Portfolio wishes to improve the reproducibility of the work that we publish. This form provides structure for consistency and transparency in reporting. For further information on Nature Portfolio policies, see our Editorial Policies and the Editorial Policy Checklist.

Please do not complete any field with "not applicable" or n/a. Refer to the help text for what text to use if an item is not relevant to your study.
For final submission: please carefully check your responses for accuracy; you will not be able to make changes later.

Statistics

For all statistical analyses, confirm that the following items are present in the figure legend, table legend, main text, or Methods section.

n/a	Confirmed	
☐	☒	The exact sample size (*n*) for each experimental group/condition, given as a discrete number and unit of measurement
☒	☐	A statement on whether measurements were taken from distinct samples or whether the same sample was measured repeatedly
☐	☒	The statistical test(s) used AND whether they are one- or two-sided<br>*Only common tests should be described solely by name; describe more complex techniques in the Methods section.*
☐	☒	A description of all covariates tested
☐	☒	A description of any assumptions or corrections, such as tests of normality and adjustment for multiple comparisons
☐	☒	A full description of the statistical parameters including central tendency (e.g. means) or other basic estimates (e.g. regression coefficient) AND variation (e.g. standard deviation) or associated estimates of uncertainty (e.g. confidence intervals)
☐	☒	For null hypothesis testing, the test statistic (e.g. *F*, *t*, *r*) with confidence intervals, effect sizes, degrees of freedom and *P* value noted<br>*Give P values as exact values whenever suitable.*
☒	☐	For Bayesian analysis, information on the choice of priors and Markov chain Monte Carlo settings
☐	☒	For hierarchical and complex designs, identification of the appropriate level for tests and full reporting of outcomes
☒	☐	Estimates of effect sizes (e.g. Cohen's *d*, Pearson's *r*), indicating how they were calculated

Our web collection on statistics for biologists contains articles on many of the points above.

Software and code

Policy information about availability of computer code

Data collection	Not applicable
Data analysis	All data analysis was performed using the publicly available SAS analytics software, version 9.4 or higher.

For manuscripts utilizing custom algorithms or software that are central to the research but not yet described in published literature, software must be made available to editors and reviewers. We strongly encourage code deposition in a community repository (e.g. GitHub). See the Nature Portfolio guidelines for submitting code & software for further information.

Data

Policy information about availability of data

All manuscripts must include a data availability statement. This statement should provide the following information, where applicable:

- Accession codes, unique identifiers, or web links for publicly available datasets
- A description of any restrictions on data availability
- For clinical datasets or third party data, please ensure that the statement adheres to our policy

Trial results are publicly accessible at the EudraCT website (https://www.clinicaltrialsregister.eu/ctr-search/trial/2015-003958-41/results). To request access to additional data, please visit https://vivli.org. Individual participant data collected during the trial and that support the research proposal will be available to qualified scientific researchers in accordance with Biogen's Clinical Trial Transparency and Data Sharing Policy on https://www.biogentrialtransparency.com. Data requests are initial reviewed by Vivli and Biogen for completeness and other parameters and are then reviewed by an Independent Review Panel. Deidentified data and study documents will be shared under agreements that further protect against participant reidentification and data are provided in a secure research environment further protecting participant privacy.

Human research participants

Policy information about studies involving human research participants and Sex and Gender in Research.

Reporting on sex and gender	All 169 participants were of the male sex. Gender was not considered in the study design. Due to the X-linked nature of choroideremia, sex-based analyses were not possible.
Population characteristics	Participants primarily identified themselves as white (144/164, 88%) and not Hispanic or Latino (131/164, 80%), had a mean (SD) baseline weight of 89.2 (17.1) kg, and were a mean (SD) age of 48.7 (13.2) years. Participants must have had active retinal pathology within the macular region and a BCVA of 34 to 73 ETDRS letters equivalent to, worse than, or equal to 6/12 or 20/40 Snellen acuity but better than or equal to 6/60 or 20/200 Snellen acuity in the study eye to be eligible for participation in the trial.
Recruitment	Participants were recruited from the natural history of choroideremia (NIGHT) study group (ClinicalTrials.gov, NCT03359551), in order to allow identification and exclusion of individuals with variable BCVA stemming from foveal splitting.
Ethics oversight	The University of British Columbia Clinical Research Ethics Board; McGill University Health Centre REB; WIRB; Columbia University IRB; John Hopkins Medicine IRB; Oregon Health and Science University IRB; UCLA IRB; HELSINKI AND UUSIMAA Ethics Committee; The Central Committee on Research Involving Human Subjects (CCMO); The National Committee on Health Research Ethics; CPP SOUTH MEDITERRANEAN V ETHICS COMMITTEE; The Ethics Committee at the Faculty of Medicine of the Eberhard-Karl University and at T0bingen University Hospital; London - West London and GTAC Research Ethics Committee

Note that full information on the approval of the study protocol must also be provided in the manuscript.

Field-specific reporting

Please select the one below that is the best fit for your research. If you are not sure, read the appropriate sections before making your selection.

☒ Life sciences ☐ Behavioural & social sciences ☐ Ecological, evolutionary & environmental sciences

For a reference copy of the document with all sections, see nature.com/documents/nr-reporting-summary-flat.pdf

Life sciences study design

All studies must disclose on these points even when the disclosure is negative.

Sample size	Sample size estimation was performed using Fischer's exact test. Considering that choroideremia is a degenerative disease, it was assumed that a ≥15-letter BCVA gain would not be observed in participants without treatment. Assuming 16.7% of the treated participants would gain ≥15 letters BCVA at 12 months, 56 participants in the high-dose group and the control group would provide ≥90% power at a 0.05 level of significance with a 2-sided test. To be conservative, 64 participants in the high-dose group and 64 participants in the control group were needed to ensure 85% power in case 1 participant in the untreated control group had ≥15-letter BCVA gain by chance, which corresponded to a total of 160 participants completing the study (64 participants in the high-dose group, 32 in the low-dose group, and 64 in the control group).
Data exclusions	No data were excluded from the analysis.
Replication	This was a Phase 3 clinical trial in which treated participants received surgical intervention. Accordingly, the results could not be tested for reproducibility.
Randomization	Randomization ratios were 2:1:2 for the high-dose, low-dose, and untreated control groups, respectively. A standard blocked randomization performed by an automated validated system was used for the random assignment of treatment and control groups.
Blinding	The requirement of a virectomy for administration of the study intervention meant a sham procedure could not ethically be performed on participants randomized to the control group. Therefore, the sponsor, investigator, and participants were all unblinded as to whether they received the intervention. However, all parties were blinded as to whether a participant received the high or low dose, and all subjective assessments were conducted by a masked assessor.

Reporting for specific materials, systems and methods

We require information from authors about some types of materials, experimental systems and methods used in many studies. Here, indicate whether each material, system or method listed is relevant to your study. If you are not sure if a list item applies to your research, read the appropriate section before selecting a response.

Materials & experimental systems

n/a	Involved in the study	
☒	☐	Antibodies
☒	☐	Eukaryotic cell lines
☒	☐	Palaeontology and archaeology
☒	☐	Animals and other organisms
☐	☒	Clinical data
☒	☐	Dual use research of concern

Methods

n/a	Involved in the study	
☒	☐	ChIP-seq
☒	☐	Flow cytometry
☒	☐	MRI-based neuroimaging

Clinical data

Policy information about clinical studies

All manuscripts should comply with the ICMJE guidelines for publication of clinical research and a completed CONSORT checklist must be included with all submissions.

Clinical trial registration

NCT03496012

Study protocol

Note where the full trial protocol can be accessed OR if not available, explain why.

Data collection

The first participant was enrolled on December 11, 2017, and the last participant completed the study on December 1, 2020. Participants across study groups were well distributed in the study sites located in Finland, Germany, the United Kingdom, and the United States.

Outcomes

The primary endpoint of the study was the proportion of participants with a ≥15-letter improvement (amended from ≥10-letter improvement, in accordance with US regulatory requirements) from baseline in BCVA at 12 months as measured by ETDRS chart. The key secondary endpoints were the mean change from baseline in BCVA at 12 months measured by the ETDRS chart, the proportion of participants with a ≥10 ETDRS-letter improvement from baseline in BCVA, and the proportion of participants with no decrease in BCVA from baseline or a decrease of <5 ETDRS letters from baseline at 12 months.

This checklist template is licensed under a Creative Commons Attribution 4.0 International License, which permits use, sharing, adaptation, distribution and reproduction in any medium or format, as long as you give appropriate credit to the original author(s) and the source, provide a link to the Creative Commons license, and indicate if changes were made. The images or other third party material in this article are included in the article's Creative Commons license, unless indicated otherwise in a credit line to the material. If material is not included in the article's Creative Commons license and your intended use is not permitted by statutory regulation or exceeds the permitted use, you will need to obtain permission directly from the copyright holder. To view a copy of this license, visit http://creativecommons.org/licenses/by/4.0/
